# Potential carriers of chemotherapeutic drugs: matrix based nanoparticulate polymeric systems

**DOI:** 10.1186/s12645-014-0003-9

**Published:** 2014-06-26

**Authors:** Dipti Kakkar Thukral, Shweta Dumoga, Shelly Arora, Krishna Chuttani, Anil K Mishra

**Affiliations:** Division of Cyclotron and Radiopharmaceutical Sciences, Institute of Nuclear Medicine and Allied Sciences, Defence Research & Development Organization(DRDO), Brig S. K. Mazumdar Road, Timarpur, Delhi 110054 India

**Keywords:** Polyethylene glycol, Crosslinkers, Methotrexate, Radiolabeling, Technetium

## Abstract

In this work matrix based nanoparticulate polymer systems have been designed using the diacrylate derivative of the well-known biocompatible polymer, poly(ethylene glycol) (PEG). This has been crosslinked using bifunctional (ethyleneglycol dimethacrylate) and tetrafunctional (pentaerythritol tetraacrylate) crosslinkers in varied concentrations (10-90%) to result in a polymeric network. The crosslinked polymers thus obtained were characterized by spectroscopic techniques (NMR and FTIR) and then prepared nanoparticles by the nanoprecipitation technique. Particle size analysis showed sizes of ~150 nm (PDI < 1) (with tetrafunctional crosslinker) and ~300 nm (with bifunctional crosslinker). Both the systems however showed unimodal narrow particle size distributions with negative zeta potential values of -15.6 and -7.3 respectively. Cytotoxicity of these formulations was evaluated by MTT assay showing non-cytotoxic nature of these carrier systems. *In vitro* drug loading and release studies were carried out using a model chemotherapeutic drug, methotrexate(MTX). These MTX loaded nanoformulations have also been evaluated biologically with the help of *in vivo* studies using radiolabeling techniques (with ^99m^Tc radionuclide). The blood kinetics profile of the formulations was studied on New Zealand Albino rabbits while the biodistribution studies were performed on balb/c mice (with EAT tumours), which revealed a hepatobiliary mode of elimination. These preliminary studies clearly demonstrated the ability of these multifunctional crosslinkers to result in tight nanosized networks with biocompatible polymers such as PEG and their potential to carry chemotherapeutic drugs.

**Electronic supplementary material:**

The online version of this article (doi:10.1186/s12645-014-0003-9) contains supplementary material, which is available to authorized users.

## Background

The past few decades have seen a tremendous advancement in the area of drug delivery using polymeric particulate carrier systems for small and large molecules. Enhanced medical treatments do not always require a stronger medicine/drug but a better mechanism to deliver the drug. Crosslinked polymeric systems have shown much interest owing to their potential applications for the entrapment and release of multitude of pharmaceutically active molecules and drugs [1]. Nanoparticulate systems are a great platform to alter and thereby help to improve the pharmacokinetic and pharmacodynamic properties of various types of drug molecules [2–4].

Matrix based nanoparticulate polymeric systems offer unique advantages for polymer based drug delivery systems (DDS) over polymer–protein conjugates [5], polymer–drug conjugates [6,7], micelles [8–10], and vesicles [11] based on amphiphilic and doubly hydrophilic block copolymers [12,13,33], dendrimers [15,16], and submicron-sized particulates [17]. These offer tunable sizes from nanometers to several micrometers and a large surface area for multivalent bioconjugation. They also have an interior network for the incorporation of therapeutics such as drugs, proteins, etc. Polymeric networks with physically entrapped bioactive molecules such as drugs, proteins, carbohydrates and DNA have been extensively investigated as targeted drug delivery carriers for biomedical applications [18]. Crosslinked polymeric systems can be prepared by chemical crosslinking of the polymeric matrices in the presence of various crosslinkers such as ethyleneglycol dimethacrylate (EGDMA), polycaprolactone (PCL) diacrylate, methyl methacrylate (MMA), divinyl benzene (DVB), pentaerythritol triacrylate (PETA), pentaerythritol tetraacrylate, etc. The crosslinking agents play a pivotal role in the preparation of nanoparticulate systems since various properties such as size, shape, structure, etc. depend on the type and concentration of the crosslinker used in the system. Such crosslinked systems show higher stability for prolonged circulation in the bloodstream owing to their chemically crosslinked structure. Their interior network acts as a drug reservoir providing enhanced drug loading by physical entrapment relative to non-crosslinked systems. The entrapment efficiency can be further enhanced by varying the crosslink density which is determined by the number of crosslinks governed by the type of crosslinker used for the preparation of the network. Drug encapsulation in these crosslinked polymeric systems such as PEG based systems could be tailored by several parameters, viz.-a-viz. the functionality of the crosslinker, the molecular weight of the PEG derivative and the architecture of the PEG molecule (linear versus star shaped PEG derivative). The improved drug loading efficiency of these systems augments the bioavailability of the drug thereby reducing any undesired side effects [19]. This is undeniably a highly advantageous aspect while using anti-cancer drugs as their side effects may result in compromised efficacy. Both biodegradable and biocompatible polymers have been investigated to prepare crosslinked networks that can carry chemotherapeutic drug payloads for therapeutic and delivery purposes. Cytarabine encapsulated chitosan nanoparticles using sodium tripolyphosphate as a crosslinking agent have been prepared that showed controlled release over a period of time [20,21]. Core-shell crosslinked nanoparticles, having polycaprolactone core and polyethyleneglycol or poly [2-(N,N-dimethylamine) ethyl methacrylate] shells have been investigated to encapsulate cisplatin and show cytostatic efficacies against a human ovarian adenocarcinoma cell line [22,23]. Extensive research has been done utilizing poly(ethylene glycol)(PEG) for drug delivery applications owing to its biocompatible nature. Cross-linked PEGs are finding increasing application as carriers of chemotherapeutic agents be it in the form of hydrogels [30,31] or in the form of copolymer micelles [32,14,34]. It was therefore proposed to use PEG as the core polymer for our work in order to further exploit its favorable attributes.

In this work we describe the synthesis and characterization of crosslinked PEG based nanoparticles and explore their potential to be used as drug delivery carriers. We used the acrylated derivative of polyethyleneglycol, i.e. polyethyleneglycol diacrylate(PEGDA) as the main polymer and crosslinked it using bifunctional and tetra functional crosslinkers, ethyleneglycoldimethacrylate (EGDMA) and pentaerythritol tetraacrylate (PETRA) respectively. The prepared nanoparticles were characterized by physico-chemical characterization techniques such as NMR, FTIR, dynamic light scattering (DLS) and Zeta potential measurements. These were loaded with methotrexate as the model chemotherapeutic agent and the drug loaded nanoparticles were then evaluated by *in vitro* and *in vivo* techniques to investigate their cellular toxicity and blood kinetics. We have also investigated their biodistribution profile in murine tumour models and imaged their tumour uptake by gamma scintigraphy post labeling with technetium-99m radioisotope (^99m^Tc).

## Methods

### Materials

Polyethylene glycol diacrylate (PEGDA) (MW 575), Pentaerythritol tetraacrylare (PETRA) and Ethyleneglycol dimethacrylate(EGDMA) were procured from M/s Sigma Aldrich, USA. Azobis(isobutyro-nitrile)(AIBN) was procured from M/s SRL, Mumbai. Technetium-99m was procured from Regional Center for Radiopharmaceuticals (Northern region), Board of Radiation and Isotope Technology (BRIT), Department of Atomic Energy, India. Infrared spectra (IR) were recorded by the KBr pellet method in the range of 4000 – 400 cm^−1^ on a Perkin Elmer Spectrum BX-II spectrophotometer. NMR measurements were carried out on a Bruker 400 MHz system. Mass spectroscopic analysis was performed using an Agilent 1100 System coupled with LC operation in ESI (Electro Spray Ionization) positive/negative mode. UV-Vis scanning was done on a UV-Vis spectrophotometer, ECIL, India, as per reported literature [24]. Gamma ray spectrometer (type GRS23C)(Electronics Cooperation of India Pvt. Ltd., India, was used for determining the amount of activity (γ-emitter) in the samples. Gamma scintillation camera (HAWKEYE) was used for imaging of animals.

#### Animal models for biological evaluation

Balb/c mice of either sex (weighing 25-30 g) with no prior drug treatment were used for biodistribution studies and gamma imaging. Healthy, New Zealand Albino rabbits (2.5-3 Kg) were employed for blood kinetics studies. All animal experiments and study protocols were approved by the Committee for the Purpose of Control and Supervision of Experiments on Animals (CPCSEA), Government of India, New Delhi.

Normal Balb/c mice, each weighing about 25-30 g, were inoculated with Ehrlich Ascites Tumour (EAT). The EAT cells were maintained in the ascites form by serial weekly passage. Exponentially growing cells were harvested and 10 ×10^6^ (approx) cells were injected subcutaneously in the right hind leg of the mice when a palpable tumour in the volume range of 0.9 ± 0.1 cm^3^ was observed in 7-10 days. Mice and rabbits were housed under conditions of controlled temperature of 22 ± 2°C and preserved on standard diet and water. All possible steps were taken to minimize the suffering of the animals at each stage of the experiment.

### Synthesis of crosslinked nanoparticles by free radical thermal polymerization

The nanoparticles were prepared by free radical thermal polymerization using AIBN as a thermal initiator. The reaction was carried out in a 3-necked round bottom flask equipped with a condenser, thermometer, round bead and a nitrogen inlet. Required amounts of the polymer (PEGDA), solvent (DMF) and the desired crosslinkers, PETRA or EGDMA, were added to the round bottom flask as per Table 1, which was placed in the water bath. As soon as the temperature of the water bath reached 70°C, AIBN was added to the reaction mixture (Table 1), and continuously stirred at a constant temperature of 70°C for 12 h. A white product precipitated out on addition of excess of cold diethyl ether which was filtered using vacuum followed by thorough washing (2-3 times) with diethyl ether. The final product was air dried overnight before further evaluation. Using the nanoprecipitation technique, nanoparticles of these crosslinked systems were prepared after optimization of the polymer concentration so as to achieve the least particle size.

**Table 1:** **Synthesis of crosslinked nanoparticles by free radical thermal polymerization**

**Formulations**	**PEGDA (% w/v)**	**EGDMA (% w/v)**	**PETRA (% w/v)**	**AIBN (% w/w)**
PE1	70	70	-----	3
PE2	70	80	-----	3
PE3	70	90	-----	3
PP1	70	-----	70	3
PP2	70	-----	80	3
PP3	70	-----	90	3

PE: EGDMA crosslinked PEGDA series; PP: PETRA crosslinked PEGDA series.

The drug loading of PP1 was carried out using MTX as a model drug in 1:10 drug:polymer ratio. The *in vitro* release profile was studied in PBS buffer (pH 7.4) and at pH 5.5 in acetate buffer. The percentage drug release was calculated using the equation:

$$ \% Drug\ released = \left[{1 - \frac{{Absorbanc{e_{(t)}}}}{Absorbance_{\sqcup_{(t0)}}}}\right] \times 100\% $$

‘t’ being the time at which the absorbance is measured and ‘t_0_’ the initial time.

#### Characterization of nanoparticles

Dynamic light scattering measurements were used to obtain values of average hydrodynamic diameter in dilute dispersions at 25°C using a Malvern Zetasizer ZS 90 equipped with a He-Ne laser beam at a wavelength of 632.8 nm (scattering angle of 90°). Prior to analysis, solutions were filtered through a Millex filter (pore size ≈ 0.45 μm) to remove dust. The determination of diffusion coefficient was calculated by fitting the data with the cumulants method and diameters were estimated by the Stokes–Einstein equation, assuming a population of non-interactive spherical particles [25].

### Cytotoxicity of nanoparticles

Cytotoxicity of the bare nanoformulations was determined using V79 fibroblast cells and the anticancer activity of the drug loaded formulation was assayed against MCF-7 breast cancer cells by the MTT {3-4,4-dimethylthiazole-2-yl)-2,5-diphenyl-2H-tetrazolium bromide} assay. Exponentially growing cells were plated in a 96-well microtitre plate at a uniform cell density of 10,000 cells/well, 24 h prior to treatment. Cells were treated with varying concentrations of the formulations (10-1000 μM) for different time intervals (12-72 h) and MTT assays were performed in triplicate. At the end of the treatment the treated cells were incubated with MTT at a final concentration of 0.5 mg/ml for 2 h at 37°C and the medium was removed. After specified time intervals (12,24,48, 72 h) the cells were lysed and the formazan crystals were dissolved using 150 μl of DMSO. Optical density was measured on 150 μl extracts at 570 nm (reference filter: 630 nm). Mitochondrial activity was expressed as percentage of viability compared to negative control. Percentage of viability = [OD(570 nm-630 nm)_test product_/OD(570 nm-630 nm)_negative control_] × 100.

### Radiolabeling of nanoformulations

Both the drug loaded and unloaded formulations were labeled with ^99m^Tc by the direct labeling method. Briefly, 100 μL of sterile sodium pertechnetate in saline (containing approx. 74-110 MBq of ^99m^TcO_4_^−^ obtained by solvent extraction method from Molybdenum) was added to 100 μL (1 mg/mL) of each formulation followed by addition of 50 μL stannous chloride (2 mg/mL in 10% glacial acetic acid); pH was adjusted to 6.5 using 0.1 M NaOH, and the resulting solution was incubated at 37˚C for 15 mins. The labeled formulations, thus obtained, were stored in sterile shielded vials for subsequent studies. The radiolabeling efficiency was determined by ascending instant thin layer chromatography (ITLC) as reported in detail elsewhere [26].

#### In vitro human serum stability

The metabolic stability of the nanoformulations was ascertained *in vitro* in freshly collected human serum from healthy volunteers. Human serum was prepared by allowing blood collected from healthy human volunteers to clot for 1hr at 37°C in a humidified incubator maintained at 5% carbon dioxide, 95% air. The samples were then centrifuged at 400 g and the serum was filtered through a 0.22 μm filter into sterile plastic culture tubes. 100 μL of ^99m^Tc labeled formulations were incubated respectively in 900 μL of this serum (in duplicate) at 37°C and analyzed to check for any dissociation of the complex by ITLC using silica gel strips and 0.9% NaCl aqueous solution (saline) as developing solvent. The change in labeling efficiency was monitored over a period of 24 hr.

### *In vivo* evaluation

#### Blood clearance and plasma protein binding studies in rabbits

Blood clearance of ^99m^Tc-labeled nanoformulations was studied in healthy New Zealand Albino rabbits weighing 2.5-3.5 kg (n = 3). 300 μL of the individual radiolabeled complexes (10 MBq) were administered intravenously through the dorsal ear vein. Blood samples were withdrawn from the other ear vein at different time intervals ranging from 5 mins-24 h. Persistence of activity in the circulation was calculated as percentage injected dose per whole blood, assuming total blood volume as 7% of the body weight. The radioactivity of the precipitate and supernatant was measured in a well-type gamma spectrometer.

Using the above blood samples, plasma was separated out by centrifugation, and the plasma proteins were precipitated by addition of 10% trichloroacetic acid (TCA). The radioactivity of the precipitate and supernatant was measured in a well-type gamma spectrometer.

#### Biodistribution in mice

Tumour bearing mice were administered 100 μL (3.7 MBq) of ^99m^Tc labeled formulations, respectively, through the tail vein(i.v.). At 1, 2, 4 and 24 h post injection, the animals (n = 3) were euthanized and blood was collected by cardiac puncture into pre-weighed tubes. The mice were then dissected and different organs (heart, lungs, liver, spleen, kidneys, stomach, intestine, bone, muscle (normal and tumour) and brain) were removed, weighed and their radioactive counts taken with the help of a gamma counter. Uptake of the radiolabeled compound into each organ was measured per gram of the tissue/organ and expressed as percentage injected dose per gram organ weight. The radioactivity remaining in the tail (point of injection) was also measured and taken into account while calculating the radioactivity.

#### Gamma scintigraphy

The tumour bearing mice were administered 100 μl (3.7MBq) of ^99m^Tc labeled formulations, respectively (i.v.), through the tail vein and gamma imaging was performed at different time intervals using a planar gamma camera equipped with a collimator.

## Results and discussion

Several criteria including control over biological, chemical and physical properties are required for the design and development of an effective nanoparticle based drug carrier system for *in vivo* biomedical applications. These may include non-toxicity to cells, stability for prolonged circulation in blood stream, high loading efficiency and controllable release of therapeutics in addition to elimination of empty carrier after drug release. With this view, an effort was made to design a nanoparticulate system based on the biocompatible PEG matrix, using PEGDA, that contains crosslinkable sites in the polymer backbone. Crosslinked nanoparticles of polyethylene glycol were prepared by free radical polymerization and crosslinking of PEGDA chains with EGDMA and PETRA in the presence of AIBN. Owing to its ease of usage we opted for the free radical thermal polymerization technique using AIBN as the thermal initiator. The minimum and maximum concentrations for forming stable particles were ranging from 70-90% for EGDMA and PETRA. Use of crosslinkers lower than 70% resulted in incomplete crosslinking while concentrations greater than 90% led to precipitation.

**Figure 1:**
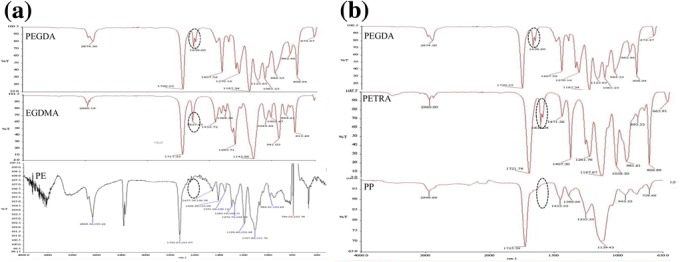
**a: FTIR spectra of PEGDA, EGDMA and crosslinked PEGDA formulations (PE).** **b:** FTIR spectra of PEGDA, PETRA and crosslinked PEGDA formulations (PP).

IR spectroscopy was performed to confirm the formation of crosslinks between PEG and the crosslinking monomers. The IR spectra of the reactants (PEGDA, EGDMA, PETRA) and the product (PEGDA-based network) were compared for changes in vital peaks (Figure 1a,b). The FTIR spectra of PEGDA, EGDMA and PETRA had typical absorption bands of medium intensity at about 1640 cm^−1^ corresponding to alkenes. This band completely disappeared in the spectra of the networks owing to the consumption of – C = C – bonds during the crosslinking reaction. Also, the wavenumber of the carbonyl stretching resonance (C = O) of the acrylate moiety shifts from 1720 to 1730 cm^−1^ on reaction. These were corroborated by the NMR spectroscopy results. The ^1^H NMR spectra of the crosslinked structures showed a complete disappearance of the peaks in the 5.5 – 6.5 ppm region corresponding to acrylate terminal functionalities of PEGDA, EGDMA and PETRA. These results indicated that the – C = C – double bonds were successfully incorporated into the PEG based network.

The average particle size of the prepared crosslinked nanoparticulate formulations was found to be in the range of 130 nm to 349 nm depending on the concentration and type of the polymeric system (Table 2). It was observed that the polymeric formulations prepared using PETRA crosslinker (PP series) were found to give relatively lower particle sizes than the ones prepared using the EGDMA crosslinker (PE series). Both the systems showed unimodal narrow particle size distributions with negative zeta potential values (Additional file 1: Figure S1 and Figure S2). The average particle size of the system consisting of EGDMA as a crosslinking agent was found to be in the range of 277 nm to 349 nm with varying concentration of the system while the particle size of the formulations varied from 130 nm to 211 nm when PETRA was used as the crosslinking agent. The zeta potential values hovered around -8 mV in the PE series whereas they varied from -13 to -21 mV in the PP series, a favorable aspect for *in vivo* applications. These results showed that as the number of the functional moiety, which is bulky acrylate group, increased from 2 to 4, the particle size of the formulations decreased by about 45-60%. This suggested that the number of the crosslinkable functional moieties strongly influences the formation of stable particles. Also, within the PE series, the particle size decreased with increasing concentration of polymer and crosslinker from PE1 to PE3, while in the PP series, the particle size increased from PP1 to PP3. That is, the smaller the number of the crosslinkable functional moieties in the crosslinking agent, the higher the concentration of crosslinking agent was needed for the formation of smaller particles. On the other hand, the larger the number of the crosslinkable functional moieties, the lower the concentration of it was needed. Similar results have been reported by other groups for the formation of crosslinked microspheres [27]. Based on these results PP1 was chosen as the model system for further drug loading experiments. But before that, it was imperative to check whether this carrier system was itself a source of toxicity to the *in vivo* system or not.

**Table 2:** **Particle size and zeta potential measurements**

**Compound**	**PE**			**Compound**	**PP**		
**Parameters**	**Size (nm)**	**PDI**	**Zeta potential (mV)**	**Parameters**	**Size (nm)**	**PDI**	**Zeta potential (mV)**
PE 1	349.0	1.00	−8.85	PP 1	130.3	1.00	−21.5
PE 2	305.0	0.48	−7.34	PP 2	154.1	1.00	−15.6
PE 3	277.5	0.51	−7.99	PP 3	211.8	0.06	−13.5

**Figure 2:**
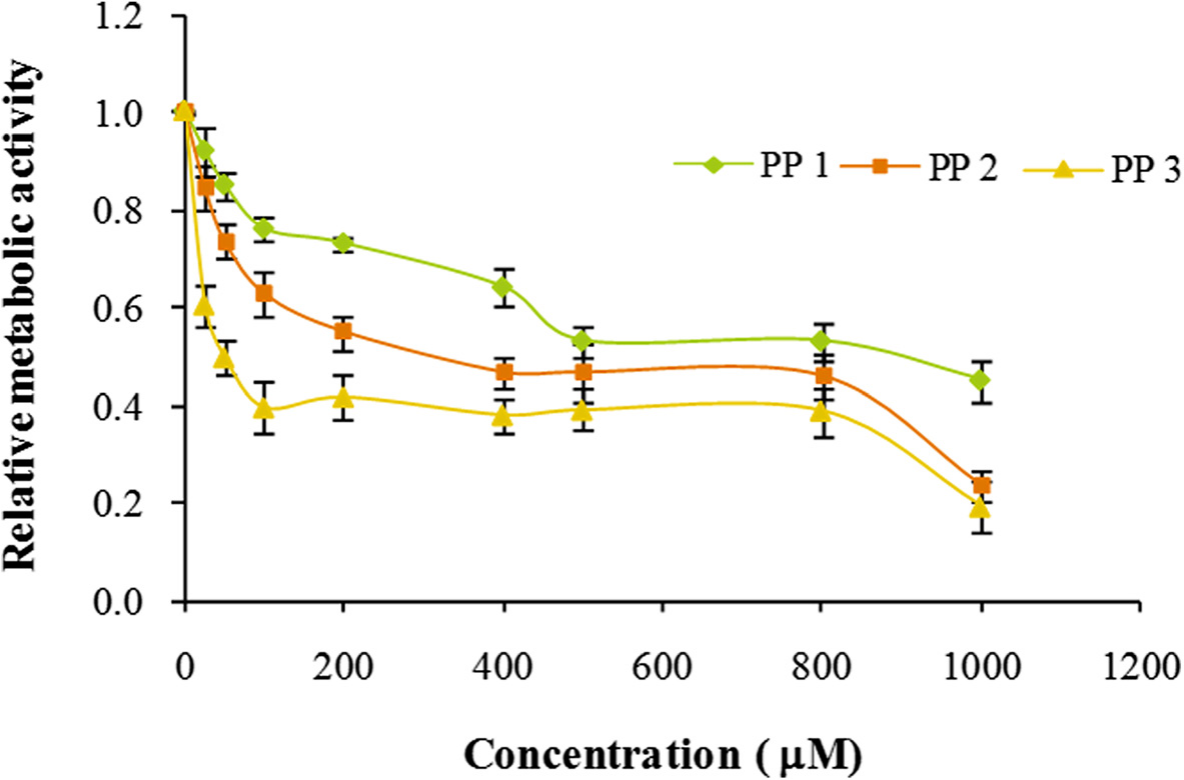
**In vitro cytotoxicity of PETRA crosslinked PEGDA formulations; PP 1, PP 2 and PP 3 after 48 h of incubation.**

**Figure 3:**
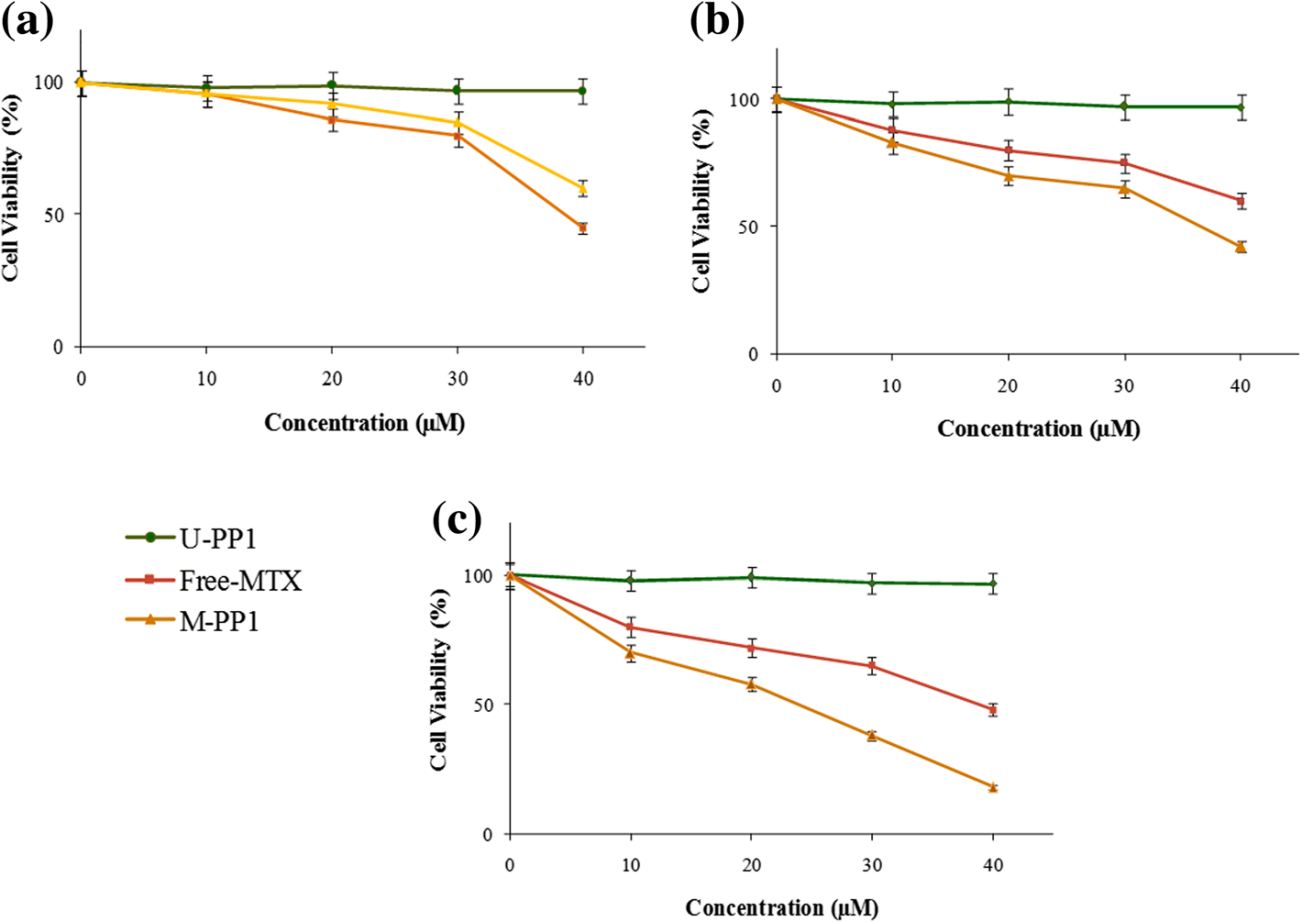
**Anticancer activity of MTX loaded PP1 in MCF-7 cell line at (a) 12 h (b) 24 h (c) 48 h.**

The *in vitro* cytotoxicity studies of this system were done by the MTT assay. The formulation was tested for its ability to induce cytotoxicity in V79 fibroblast cells using an assay of mitochondrial activity (MTT assay). After 24, 48, and 72 h of exposure, viability was assessed on the basis of cellular conversion of MTT into a formazan product. A plot of percentage viability against the concentration revealed a decrease in the mitochondrial activity of the nanoformulation with increased concentration of upto 1000 μM (Figure 2). Even after 48 h of incubation, the formulations appeared to be non-toxic upto very high concentrations of almost 800 μM. These results suggested that the PETRA crosslinked nanoparticles of PEGDA are nontoxic to normal cells which confirmed their safety for non-cancerous cells. Of these PETRA crosslinked formulations, PP1, which formed the smallest size of nanoparticles, was taken as the matrix based system for further drug loading and biological evaluations. The anticancer activity of the free MTX and MTX loaded PP1 (M-PP1) was studied on MCF-7 breast cancer cells. MTT assay showed that both the free drug and the drug loaded nanoformulation could significantly suppress MCF-7 cell proliferation in a dose and time dependent manner (Figure 3a-c). While at the same concentration, the MTX loaded PP1 showed significantly higher inhibition rates against MCF-7 cells compared to free MTX especially at higher time points of 24 h and 48 h. In this the unloaded nanoformulation (U-PP1) was taken as the control and it was found to show negligible effect on cell viability (Figure 3). Thus the cytotoxicity of the MTX loaded nanoformulations was confirmed to be because of the encapsulated MTX.

**Figure 4:**
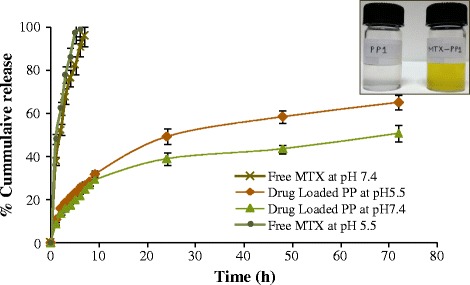
**In vitro Drug release profile of free MTX and MTX loaded PETRA crosslinked PEGDA formulation over 72h.**

Polymeric nanoparticles can be loaded with drugs using three major strategies viz.-a-viz. covalent attachment of the drug to the particle surface or to the polymer prior to preparation, adsorption of the drug to a preformed carrier system, and incorporation of the drug into the particle matrix during particle preparation [28]. However, the important condition is that the bioactivity of therapeutic agents must remain intact during this preparation process. With this in view the crosslinked PEG network was used to encapsulate MTX. By optimization of the nanoprecipitation conditions our PEG based crosslinked matrix readily self assembled into reproducible polymeric micelles actively encapsulating the MTX particles in solution. The MTX loaded dispersions (MTX-PP1) displayed a yellow colour as compared to the almost translucent unloaded ones (Figure 4 inset). In order to study the *in vitro* release of the incorporated drug, membrane diffusion techniques are often used. In these cases, drug release follows more than one mechanism. In case of release from the surface of the nanoparticles, the adsorbed drug quickly dissolves on contact with the release medium. The entrapped drug then gets gradually released by the diffusion mechanism. Loading and release characteristics of methotrexate from these particles was studied at pH 5.5 and 7.4 using these membrane diffusion techniques. The unloaded drug and the organic solvent DMSO were removed by dialysis against distilled water. Drug loading content and drug loading efficiency was then determined spectrophotometrically at 303 nm. The MTX loaded PP1 was found to have a drug loading content of 7.68% and an encapsulation efficiency of 65.8%. The drug release profile of free MTX and MTX loaded polymeric nanoparticles over a 72 h study period is shown in Figure 4. Free MTX was completely released within 6 h at pH 5.5 and within 7 h at pH 7.4 while after encapsulation in the PEG based matrix the *in vitro* release pattern of MTX showed a drastic change and only 20-25% of the drug could be released from the matrix bound particles within the same duration. This clearly indicates the absence of any free unloaded MTX in the prepared polymeric particle solutions as no burst release effect could be seen. The release studies of the MTX-loaded particles indicate a biphasic release pattern, similar for both the pH conditions. Almost 40-50% of the drug was released from the particles within the first 24 h. Thereafter, a very slow drug release pattern was observed and a cumulative 50-65% MTX release was seen over a period of 72 h (the period of study) owing to the release of the entrapped drug by the diffusion mechanism. This could be an advantageous feature of this MTX-loaded polymeric system where initial fast drug release provides a higher dose followed by a controlled release during the rest of the duration of therapy.

PP1 and the MTX loaded PP1 formulations (MTX-PP1) were labeled with ^99m^Tc. All the labeling parameters such as pH, concentration of reducing agents (SnCl_2_), temperature, etc. were standardized to achieve the maximum labeling efficiency [29]. The proteolytic degradation of the radiolabeled formulations was determined in human serum *in vitro*. ITLC analysis of the human serum revealed that the ^99m^Tc-nanoformulations remained sufficiently stable during incubation at 37°C with human serum. A maximum of 8% of radioactivity degraded after 24 h of incubation advocating a high *in vitro* stability of almost 92% of the nanoformulation for upto 24 h.

**Figure 5:**
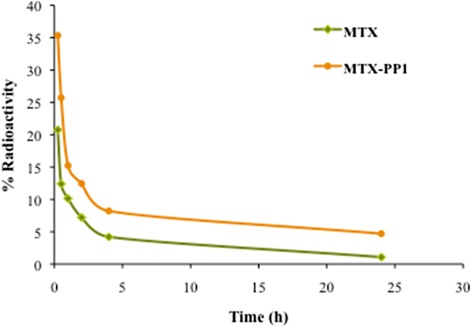
**Blood clearance profile of** ^**99m**^ **Tc labeled MTX and PETRA crosslinked PEGDA formulation (PP1) over a period of 24 h.**

A comparative evaluation of the blood clearance profile of the radiolabeled drug and nanoformulation in normal rabbits showed a biphasic mode of clearance (Figure 5). The radiolabeled drug, ^99m^Tc-MTX demonstrated a quicker washout from the blood in a biphasic manner with a fast and slow half-life time of 35 mins and 17 h respectively, and only 25% of the injected dose remaining after 2 h of injection. However, the radiolabeled nanoformulation, ^99m^Tc-MTX-PP1, exhibited relatively slower clearance in a somewhat biphasic pattern from blood circulation as expected for a matrix based system with almost 50% of the injected labeled compound remaining in blood 1 h post injection. The rest remained in circulation for upto 24 h and was gradually cleared from systemic circulation owing to the small size of the formulations and also perhaps not being readily taken up by the reticuloendothelial system (RES). 75-85% of the nanoparticulate carriers were eliminated from the blood circulation within 24 h. The t_½_ (fast) for the nanoformulation was found to be 45 mins, and the t_½_(slow) was 27.5 h respectively. At every time point the amount of the labeled MTX-PP1 conjugate in blood was more than the neat drug, thus suggesting longer blood retention, as also indicated by the half life-time values.

Protein binding studies showed a difference in the plasma protein binding of ^99m^Tc-MTX as compared to its encapsulated form. Binding of ^99m^Tc-MTX to plasma proteins was only 68% whereas it was 54-58% in the case of ^99m^Tc-MTX-PP1 nanoformulation probably because of the PEG based matrix.

**Figure 6:**
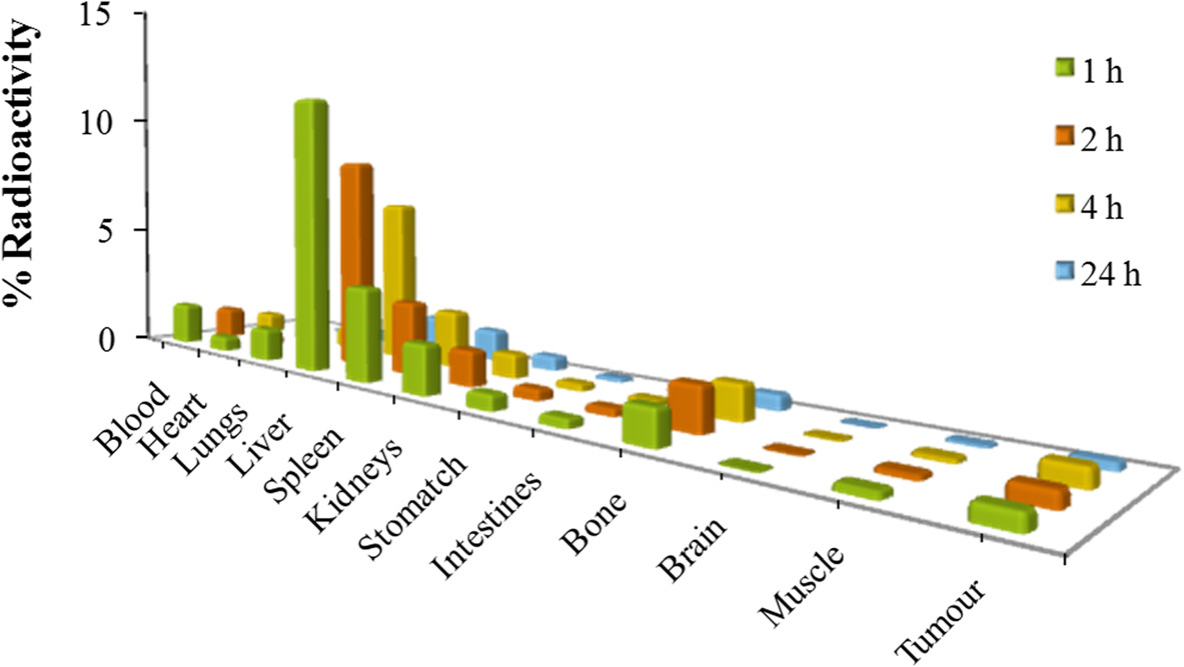
**Biodistribution of MTX loaded PETRA crosslinked PEGDA formulation (PP1) over a period of 24 h.**

**Figure 7:**
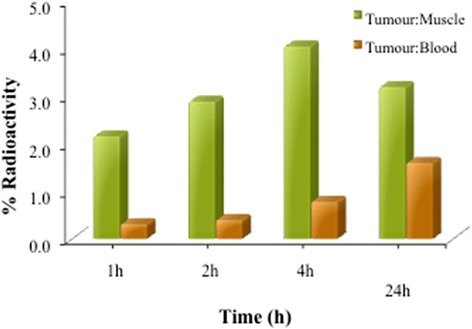
**The relative tumour uptake of PETRA crosslinked PEGDA formulation (PP1) compared to the contralateral normal muscle (tumour:muscle ratio) over a period of 24 h.**

Figure 6 represents complete tissue distribution after intravenous administration of ^99m^Tc-labeled MTX-PP1 and results are expressed as percentage of the injected dose of ^99m^Tc-labeled compounds accumulated per gram of tissue (%ID/g of tissue) at different time intervals. Biodistribution study of the drug loaded formulation in tumour bearing mice showed remarkable localization in tumour muscles (Figure 6) which could be well differentiated as compared to the negligible uptake in the normal muscles. This was also corroborated by the tumour-to-blood ratios that improved at increasing time points (Figure 7). A low uptake of radioactivity was observed in the stomach at all the time points(less than 2% ID/g) indicating a minimal *in vivo* decomposition of the radiolabeled nanoformulation to form the free ^99m^TcO_4_^−^. The radiolabeled formulation accumulated to a higher level in the liver and kidneys at early time points after injection. The route of excretion of both the compounds was therefore perceived to be hepatobiliary. This clearly indicated that these crosslinked nanoparticulate carriers eventually get eliminated from the body.

**Figure 8:**
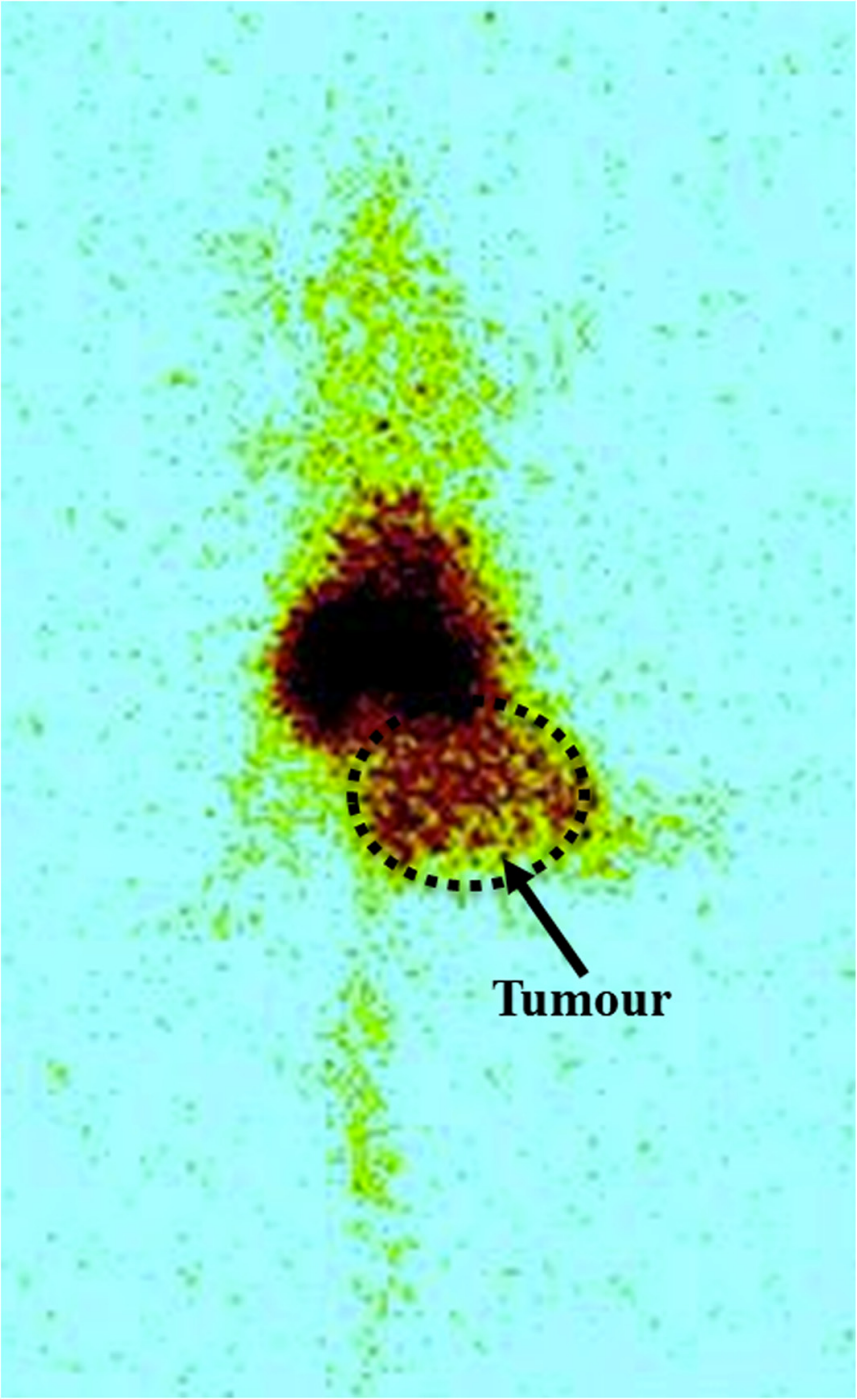
**Whole-body γ-scintigraphic image of balb/c mice with subcutaneous EAT tumor above the right hind leg injected with** ^**99m**^ **Tc labeled PETRA crosslinked PEGDA formulation.**

The relative tumour uptake of the ^99m^Tc labeled formulations compared to the contra-lateral normal muscle (tumour:muscle ratio) in balb/c mice is shown in Figure 7. The MTX loaded formulation showed high uptake in tumour muscles. This uptake helped to clearly delineate the tumour from the rest of the normal muscle which was also corroborated by the whole body tumour scintigraphic image shown in Figure 8.

## Conclusion

Synthesis and characterization of the PEG based nanoparticles has been described in this study. These were successfully loaded with the anticancer drug, MTX, to evaluate their potential for anticancer drug delivery. The *in vitro* drug release pattern of the drug showing an initial fast release within the first 6 h followed by an eventual gradual release over 72 h could well be exploited for therapy regimes spread out over long durations thereby reducing the frequency of drug uptake. For clinical relevance, in addition to obtaining low non-specific uptake in healthy cells, sufficient uptake must occur in tumour cells to cause cell death. The MTT assay on fibroblast cells (healthy cells) and MCF-7 cells(human carcinoma) proved the clinical significance of these MTX loaded PETRA crosslinked PEGDA nanoparticles. The avid tumour uptake of the ^99m^Tc-labeled nanoformulation and its gradual tumour washout demonstrated its use both as a tumour diagnostic agent as well as a therapeutic agent for cancer therapy. However, detailed studies on tumour proliferation rate and survival rates in animal models can give significant information on the therapeutic efficacy of these particles, which can form part of our future research work. Also, labeling of these crosslinking polymeric systems with gadolinium as an imaging marker can also form part of our future studies so imaging with MRI can be utilized. The investigations carried out as part of this work gave adequate evidence that the prepared crosslinked polymeric nanoparticulate formulations may be vital nanoparticulate carriers of chemotherapeutic drugs such as methotrexate, for potential drug delivery applications.

## Additional file

Additional file 1:
**Figure S1 and Figure S2.** Gives details of the size and zeta potential measurements of EGDMA and PETRA crosslinked PEGDA formulations respectively.
