# Supplementary material for: Potential carriers of chemotherapeutic drugs: matrix based nanoparticulate polymeric systems
Source: Cancer Nanotechnol. 2014 Jun 26;5(1):3. doi: 10.1186/s12645-014-0003-9 (PMC4631724; doi:10.1186/s12645-014-0003-9)
Supplement: Additional file 1: — Figure S1 and Figure S2. Gives details of the size and zeta potential measurements of EGDMA and PETRA crosslinked PEGDA formulations respectively. [file 12645_2014_3_MOESM1_ESM.pptx]

## Slide 1
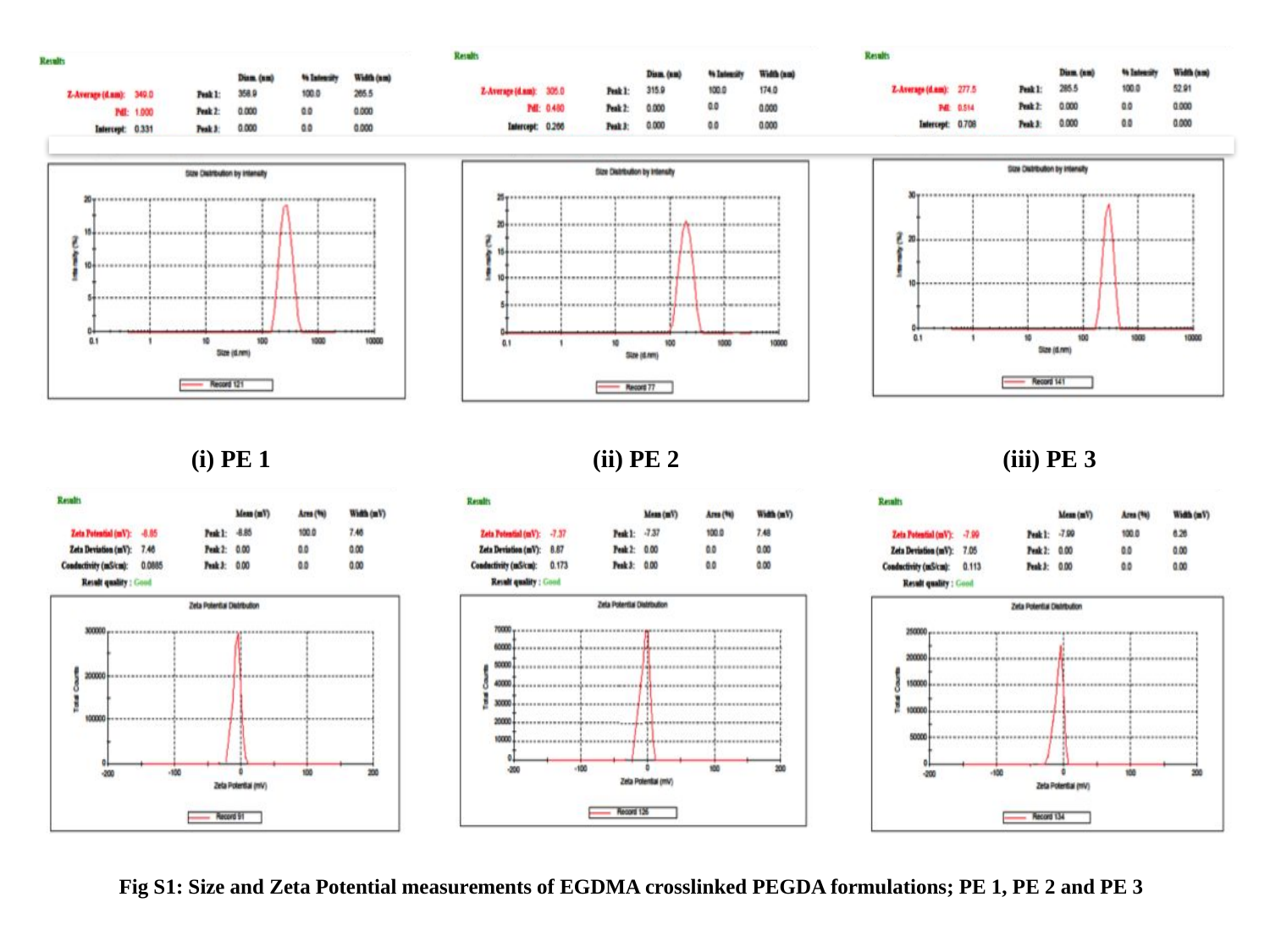

(ii) PE 2
(iii) PE 3
(i) PE 1
Fig S1: Size and Zeta Potential measurements of EGDMA crosslinked PEGDA formulations; PE 1, PE 2 and PE 3

## Slide 2
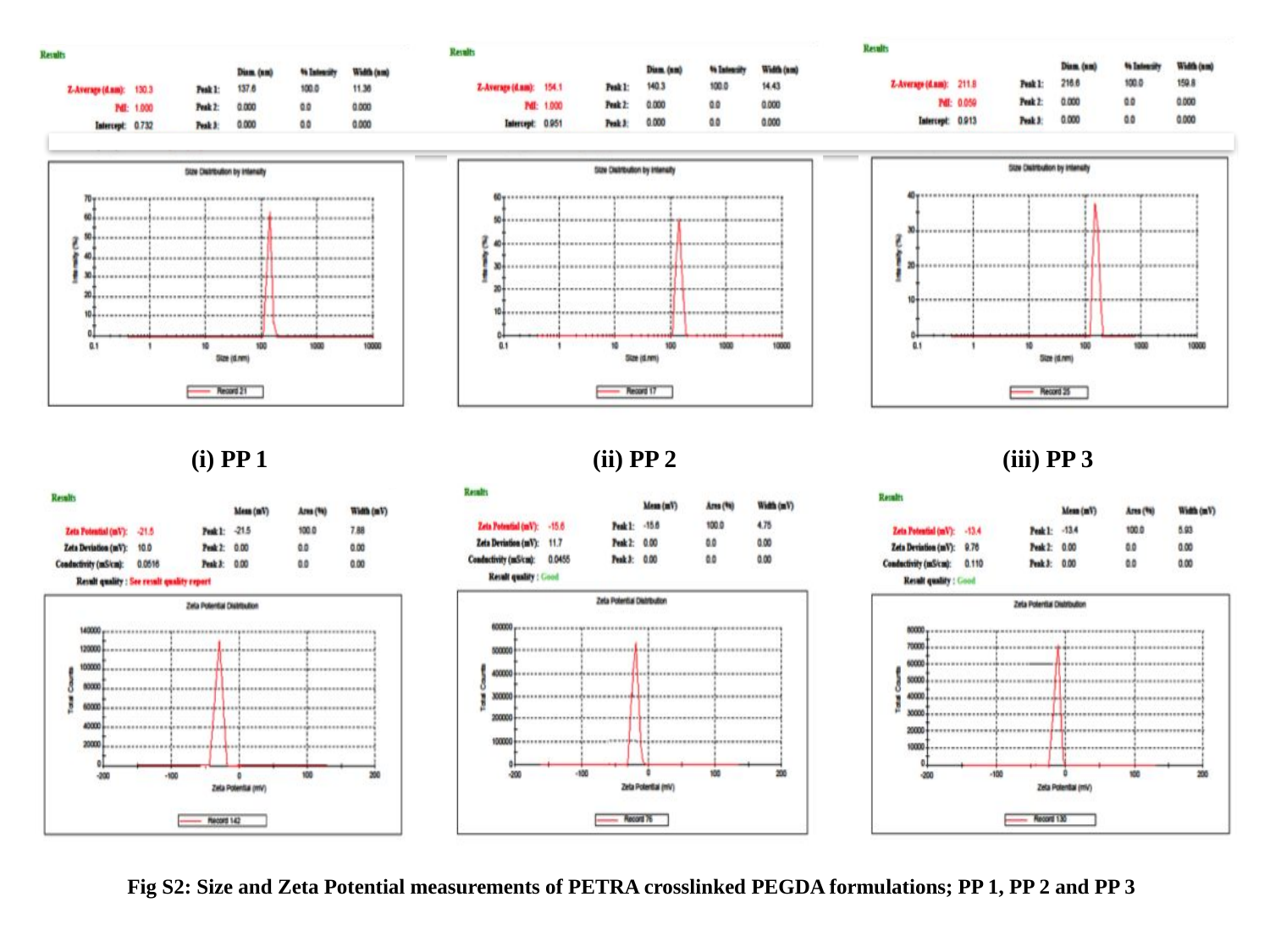

(ii) PP 2
(iii) PP 3
(i) PP 1
Fig S2: Size and Zeta Potential measurements of PETRA crosslinked PEGDA formulations; PP 1, PP 2 and PP 3
